# Wales’ Objectives

**Published:** 1868-11

**Authors:** 


### WALES' OBJECTIVES.

Within the past few months we have been so fortunate as to add to our microscopic appliances two of Wales best high power objectives—a $\frac{1}{15}$ and $\frac{1}{30}$, both working either dry or by immersion, better by the latter than the former, as by it greater working distance and better definition are obtained.

We do not propose here to attempt to give a description of the powers or capabilities of these glasses, but will simply state that in the limited use we have been able to make of them, we have obtained results which we had not been able to approximate before, and we have been using some very excellent objectives.

In the structure of dentine they reveal many things that we had not before witnessed or seen described by others. It will afford us much gratification if in the future, circumstances will permit, to present to the readers of the REGISTER some observations, on the structure of dentine as revealed by these glasses.

We fully endorse so far as our observation extends, the encomiums that have been attached by some of our best microscopists to Mr. Wales' instruments.

Perhaps when we have more time and knowledge, we will say more upon this subject. T.

